# Best prediction for need of dialysis following cardiac surgery is obtained with the Thakar model

**DOI:** 10.1186/cc9532

**Published:** 2011-03-11

**Authors:** HD Kiers, MC Schoenmakers, HA Van Swieten, JG Van der Hoeven, S Heemskerk, P Pickkers

**Affiliations:** 1 Radboud University Nijmegen Medical Centre, Nijmegen, the Netherlands

## Introduction

Postoperative acute kidney injury requiring dialysis (AKI-D) occurs in 1 to 5% of patients after cardiac surgery with cardiopulmonary bypass (CPB) and is associated with a high mortality (30 to 60%) and prolonged increased ICU length of stay. There are four models using different covariates that aim to predict the risk for postoperative AKI-D in cardiac surgery patients [1-4]. We aim to investigate which model best predicts AKI and AKI-D in our cardiac surgery population.

## Methods

All adult patients undergoing cardiac surgery with CPB, between October 2006 and January 2009, in our hospital were included in this study. Data on preoperative risk factors and postoperative changes in serum creatinine levels of all patients were collected with the use of hospital databases and medical records. AKI was defined according to RIFLE (Risk, Injury, Failure, Loss and End-stage Kidney Disease). AKI-D was defined as the need for hemodialysis during the first 6 days following cardiac surgery. We assessed the discrimination of each model using the area under the curve of the receiver operating characteristics (AUC-ROC, see Table 1) curve for prediction of AKI and AKI-D.

**Table 1:** AUC-ROC for four models for the prediction of AKI-D and AKI

Model	*n*	AKI-D (95% CI)	AKI (95% CI)
Chertow	918	0.80 (67 to 93)	0.65 (58 to 72)
Thakar	928	0.95 (90 to 99)	0.77 (70 to 83)
Mehta	866	0.81 (66 to 96)	0.74 (67 to 81)
Wijeysundera	924	0.93 (90 to 97)	0.73 (67 to 80)

## Results

A total of 966 patients were included in this study, of which 926 medical records were available for review. The procedures performed were coronary artery bypass grafting CABG (*n *= 733, 79%), single valve surgery (*n *= 79, 9%) or CABG and valve or other surgery (*n *= 114, 12%).

The median change in serum creatinine was +6% (IQR -24% to +17%) during the first 6 days after surgery. AKI developed in 32 (3.4%) and in 19 (2.0%) patients classified as Risk and Injury, respectively. AKI-D developed in 13 (1.7%) patients. Table 1 shows the AUC-ROC curve value for each model (*P *< 0.001 for all data) for the prediction of AKI and AKI-D.

## Conclusions

The model of Thakar is the best predictor of AKI and AKI-D in our population.
